# Maternal education and adolescent psychological symptom severity: the synergistic reversal trade-offs between perceived maternal support and parental educational expectations

**DOI:** 10.3389/fpsyt.2026.1858459

**Published:** 2026-07-24

**Authors:** Cui Xu, Yiwen Zhang, Nianyu Du, Huiying Peng, Shijiu Yin

**Affiliations:** 1 School of Statistics and Data Science, Qufu Normal University, Qufu, China; 2 International College, Yunnan Agricultural University, Kunming, China; 3 School of Economics, Qufu Normal University - Rizhao Campus, Rizhao, China; 4 School of Health and Elderly Care, Shandong Women’s University, Jinan, China

**Keywords:** maternal education, adolescent psychological symptom severity (APSS), inverted U-shaped curve, synergistic reversal trade-offs, Database of Youth Health

## Abstract

**Background:**

Adolescence is a critical period with a high incidence of psychological and behavioral problems. In China, adolescent mental health presents a severe challenge. The family serves as the core environment for adolescent development, within which maternal education is a key factor influencing adolescent mental health.

**Methods:**

Based on the data from the “Database of Youth Health” (DYH), this study employed a cross-sectional design and used a quadratic regression model to examine the impact of maternal education on adolescent psychological symptom severity (APSS), with a focus on testing for non-linear effects. The robustness of the findings was verified through a U-test for non-linearity and an instrumental variable approach. Additionally, mediation and moderation analyses were conducted to explore the underlying mechanisms and heterogeneity involved.

**Results:**

Regression analysis revealed an inverted U-shaped relationship between maternal education and APSS (higher SCL-90 scores indicate greater severity), with the peak occurring at 6.442 years of maternal education. This non-linear relationship emerged from synergistic reversal trade-offs between perceived maternal support and parental educational expectations. Heterogeneity analysis indicated that before the educational inflection point, non-only children exhibited significantly lower APSS, whereas beyond this point, only children demonstrated a steeper reduction in symptom severity.

**Conclusion:**

This study reveals an inverted U-shaped relationship, driven by a “synergistic reversal trade-off” mechanism: as maternal education increases, both perceived maternal support and parental educational expectations simultaneously shift from risk to protective factors. The dual-pathway reversal is amplified in only-child families, revealing steeper risks at low education levels and stronger protective benefits at high levels. These findings offer empirical evidence for differentiated, mechanism-driven family interventions.

## 1 Introduction

Adolescence^1^ is a critical developmental stage characterized by a high incidence of psychological and behavioral problems (1). Research indicates that approximately 50% of lifetime mental illnesses have their onset by age 14 (2), and mental health during this period exerts a profound influence on an individual’s lifelong development (3). Currently, the mental health status of adolescents in China presents a concerning picture. It is estimated that about 25% of Chinese adolescents exhibit mild to severe depressive symptoms, and over 30 million individuals under the age of 17 are affected by emotional or behavioral disorders (4).This severe situation necessitates urgent attention and effective intervention from all sectors of society.

In response to this adolescent mental health crisis, China has initiated a systematic policy-level response. The “People’s Republic of China Family Education Promotion Law” (5) explicitly designates the family as the “primary responsible entity” for mental health education, emphasizing the pivotal role of parents as “first teachers”. This legislative orientation aligns with ecological systems theory (6), which underscores the family’s central role in adolescent development. Empirical research, such as that by Reiss, demonstrates that parental education is a key determinant of adolescent mental health (7). Parental education level not only reflects a family’s cultural and educational resources but also signifies its capacity to translate health awareness into concrete practices (8). Within the Chinese familial context, mothers often assume the role of “significant others” in adolescent socialization due to traditional gender-based divisions of labor (9, 10). Consequently, maternal education emerges as a particularly critical focal point for efforts aimed at promoting adolescent mental health.

Regarding the relationship between maternal education and adolescent mental health, existing research shows significant divergence in perspectives. Some scholars argue that mothers with higher education levels promote adolescent mental health by optimizing parent-child interactions and adopting evidence-based parenting styles (e.g., higher expectations but less harshness) (9–11). In contrast, other studies suggest that mothers with higher education levels may exert greater academic pressure (12), thereby increasing mental health risks for adolescents. Additionally, from the perspective of ecological systems theory, as adolescents age and other environments (such as peers and schools) become more influential, the impact of parental education may diminish (9). Some studies propose that in specific contexts (e.g., Asian regions or low- and middle-income countries), there is no significant association between the two (10). These discrepancies in findings can be attributed to two main research limitations: First, most existing studies are based on linear assumptions, overlooking the possibility of nonlinear relationships. For example, the protective effect of maternal education on mental health may be stronger in younger years and weaken over time, indicating a developmental nonlinearity (9). Second, existing mechanism research is fragmented and fails to adequately incorporate the interactions between distal resources (e.g., maternal education) and proximal processes (e.g., parental support and educational expectations) proposed by ecological systems theory (9, 13), making it difficult to comprehensively explain how distal resources influence adolescent mental health through proximal processes.Therefore, this study aims to address two key questions: Does maternal education level have a nonlinear impact on adolescent mental health? If so, what are the mechanisms?

This study draws on ecosystem theory and social capital theory to construct a “distal resources–proximal processes” analytical framework. Utilizing cross-sectional data obtained through a PPS sampling method from the DYH, it investigates the impact of maternal education on APSS and its underlying mechanisms.To identify the causal effect of maternal education on APSS, we specify a baseline econometric model that incorporates a quadratic term. We then conduct a series of robustness checks to validate our findings. These include a U-test for nonlinearity and an instrumental variable (IV) approach, using the average years of maternal education at the city level as the instrument. Multiple estimation models—Two-Stage Least Squares (2SLS), Limited Information Maximum Likelihood (LIML), and Generalized Method of Moments (GMM)—are employed to ensure the robustness of the IV estimates. Furthermore, the study examines the mediating roles of perceived maternal support and parental educational expectations through a mediation analysis. Finally, we analyze potential heterogeneity by testing the moderating effect of being an only child, which is implemented by incorporating relevant interaction terms into the model.

This study makes three primary contributions. First, empirically, it moves beyond the linear assumption and reveals a significant inverted U-shaped relationship between maternal education and APSS. This finding provides novel empirical evidence on the complex influence of advantageous family resources, thereby challenging the prevailing view of a monotonically positive effect.

Second, regarding mechanisms, this study integrates ecosystem theory and social capital theory to construct a “distal resources–proximal processes” analytical framework. It uncovers a synergistic reversal trade-off between two mediating pathways: perceived maternal support and parental educational expectations. In this trade-off, both pathways simultaneously reverse their direction from risk to protective factors as maternal education increases. This provides a precise, micro-level mechanistic explanation for the observed inverted U-shaped relationship. Consequently, it advances the literature from testing “whether an effect exists” to explaining “the dynamic process through which the effect is constituted.

Third, theoretically, drawing on bioecological model (14), the study identifies a conditional moderating role of being an only child, where the direction of moderation varies with maternal education level. This finding deepens the “resource dilution” theory by showing that the dilution effect depends on the abundance of family resources themselves. It thus offers a new perspective for understanding the interaction between family structure and resource transmission in times of rapid social change.

## 2 Theoretical analysis and research hypotheses

### 2.1 The impact pathways of maternal education on APSS: supportive and expectation pathways

The Bioecological Model defines the core driving mechanism of human development as proximal processes—enduring, reciprocal interactions between the developing person and persons, objects, and symbols in their immediate environment (6). Within this model, mothers are widely recognized as the most influential “significant others” in adolescents’ family microsystems (9, 10). Thus, maternal education, as a key distal contextual resource, indirectly affects adolescent mental health by shaping proximal processes within the family microsystem (9).

To specify the concrete dimensions of these proximal processes, we draw on Coleman (15) social capital theory, which posits that family social capital facilitating intergenerational human capital transfer operates through two key forms: information networks (e.g., quality of emotional/resource support) and responsibility expectations (e.g., educational achievement expectations). Existing studies confirm that differences in maternal education significantly affect the quality of support provided and educational expectations set (16). Thus, maternal education influences APSS through two proximal pathways: the supportive pathway (perceived maternal support, an information network resource) and the expectation pathway (parental educational expectations, a responsibility expectation resource).

### 2.2 The dual-pathway mechanisms: support and expectations

#### 2.2.1 Mediating effect of perceived maternal support

At lower maternal education levels (below the inflection point), mothers face human capital constraints, such as limited time, parenting knowledge, and communication strategies, which result in less structured and less cognitively stimulating parenting behaviors (17). Consequently, adolescents are more likely to perceive these behaviors as interference or control rather than support (18). This negative perception, coupled with the generally low quality of proximal processes in low-education families, is associated with increased APSS (9). At the inflection point, this risk pathway begins to attenuate. Above this threshold, higher maternal education is associated with more structured, cognitively stimulating parenting styles characterized by open communication and guided support (19, 20). These high-quality proximal processes, by buffering psychological stress, foster the positive perception of maternal support, thereby predicting lower APSS (9, 10).

#### 2.2.2 Mediating effect of parental educational expectations

Maternal education influences adolescent mental health by affecting both the absolute level of educational expectations and parent-child academic socialization communication. Research has shown that parents’ own socioeconomic status, particularly their educational level, is significantly positively correlated with their educational expectations for their children (21). At lower levels of maternal education, although parental educational expectations rise with maternal education, they remain relatively low in absolute terms. Low parental educational expectations are often accompanied by less academic socialization communication—such as discussing the future and setting goals (17). This may lead to a “parent-child consistent low expectations” developmental pattern. As a result, adolescents lacking clear goals and external norms are more prone to behavioral problems and low self-esteem, thereby increasing APSS (22). At higher levels of maternal education, parental educational expectations increase significantly. When these high expectations are transmitted through academic socialization, they can satisfy adolescents’ basic psychological needs and enhance academic engagement, thereby directly and indirectly reducing APSS (17).

#### 2.2.3 Analysis of the synergistic reversal trade-offs between perceived maternal support and parental educational expectations

The preceding sections have respectively elaborated how the supportive pathway and the expectation pathway independently influence APSS. Through the dynamic matching between perceived maternal support and parental educational expectations, these two pathways synchronously reverse their effects, ultimately driving the inverted U-shaped relationship between maternal education and APSS.

Kahneman and Tversky (23) reference point theory suggests that an individual’s judgment and emotional response depend on the discrepancy between the actual state and a reference point. In the family context, maternal education shapes the objective resource environment of the family, which in turn influences adolescents’ subjective perception of maternal support (the actual state), while parental educational expectations constitute the key psychological reference point. Adolescents compare their “perceived maternal support” with “parental educational expectations.” When perceived support falls below parental educational expectations, a perception of loss and loss aversion arises, directly exacerbating psychological symptoms. Conversely, when perceived support matches or exceeds expectations, a perception of gain emerges, alleviating psychological symptoms.

Therefore, the direction of the effects of both pathways is ultimately dynamically governed by the same background condition: the relative relationship between “perceived maternal support” and “parental educational expectations” (i.e., whether they are matched or mismatched). It is precisely this change in the “perceived maternal support and parental educational expectations match” background that determines whether each pathway manifests as a risk or a protective factor. At the low maternal education end, perceived maternal support is lower than parental educational expectations, and both pathways simultaneously manifest as risks. At the high maternal education end, perceived maternal support matches or exceeds parental educational expectations, and both pathways simultaneously manifest as protection, thereby synergistically generating the inverted U-shaped relationship.

### 2.3 The moderating role of family resource concentration: the only-child effect

The bioecological model points out that the form, intensity, content, and direction of proximal processes are systematically regulated by the characteristics of the developing individual (14). This study treats only-child status as a personal demand characteristic: resources and parental attention are highly concentrated in only-child families, which may alter the overall effect pattern of maternal education on adolescent psychological symptoms. Based on the resource dilution theory, we expect only-child status to amplify the strength of the inverted U-shaped relationship: higher risks at the low-education stage and stronger protective effects at the high-education stage. Therefore, we further add the interaction terms of only-child status with the linear and quadratic terms of maternal education in the benchmark regression model to test whether only-child status moderates the inverted U-shaped relationship between maternal education and APSS.

Accordingly, we propose the following research hypotheses:

- H1: There is an inverted U-shaped relationship between maternal education and APSS.
- H2a: The indirect effect of maternal education on APSS through perceived maternal support is positive (risk-inducing) at low education levels and negative (protective) at high education levels.
- H2b: The indirect effect of maternal education on APSS through parental educational expectations is positive (risk-inducing) at low education levels and negative (protective) at high education levels.
- H2c: The synergistic reversal trade-offs between perceived maternal support and parental educational expectations (as described in H2a & H2b) collectively produce an inverted U-shaped total indirect effect of maternal education on APSS.
- H3: Only-child status moderates the inverted U-shaped relationship between maternal education and APSS, making the inverted U-shaped curve steeper for only-child groups than for non-only-child groups (i.e., higher risks at the low-education stage and stronger protective effects at the high-education stage).

**Figure 1:**
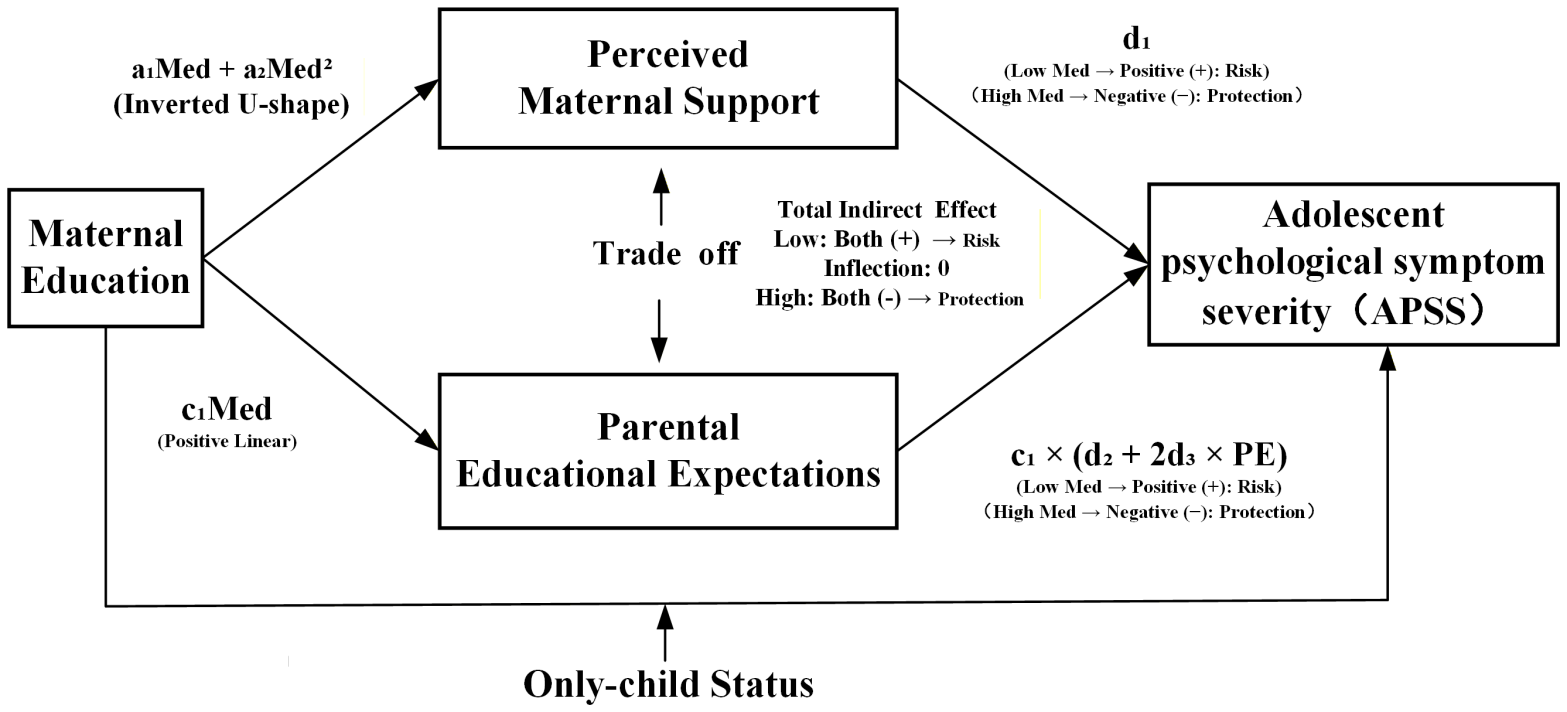
Schematic diagram of the theoretical mechanism by which maternal education influences APSS. APSS was measured by the Symptom Checklist-90 (SCL-90); higher scores indicate greater symptom severity.

Based on the analysis, this paper proposes a theoretical hypothesis model as illustrated in **Figure 1**.

## 3 Materials and methods

### 3.1 Data and participants

This study’s data were derived from the Database of Youth Health (DYH)^2^, compiled by Shandong University. The database utilizes a repeated cross-sectional design, incorporating 186 junior and senior high schools across 17 cities in Shandong Province. The sampling methodology of DYH involves stratification based on the geographical distribution, population density, and socioeconomic levels of Shandong Province, following the principle of probability proportional to size (PPS) sampling to select sample schools. Small-scale schools were excluded, specifically those with an average of fewer than 100 students per grade or a total enrollment below 300. Primary data were collected through standardized questionnaires, supplemented by on-site physical fitness assessments and archival data retrieval. These data encompass core health indicators including adolescent physical fitness, mental health, nutritional diet, and social behavior. After preprocessing procedures such as outlier detection, handling of missing values, and coding and merging of variables, a final analytic sample of 3, 423 valid observations was obtained.

### 3.2 Measures

#### 3.2.1 Dependent variable

Adolescent psychological symptom severity (APSS) was assessed using the Symptom Checklist-90 (SCL-90), developed by Derogatis et al. (24). Higher total SCL-90 scores indicate greater symptom severity. This scale comprises 90 items across nine dimensions, including somatization and obsessive-compulsive symptoms, each rated on a 5-point Likert scale (1 = none to 5 = severe). The scale has demonstrated strong psychometric properties among Chinese adolescents (Cronbach’s α = 0.97) (25). A total SCL-90 score of ≥160 was used as the positive criterion for psychological abnormality.

#### 3.2.2 Key independent variable

Maternal education (Med) was measured as years of schooling, converted from the mother’s highest educational attainment as follows: no schooling = 0 years, primary school = 6, junior high school = 9, high school/technical secondary school = 12, associate’s degree = 15, bachelor’s degree = 16, and master’s degree = 19. To capture the hypothesized non-linear effects, both the linear term (Med) and its squared term (Med^2^) were included in the model.

#### 3.2.3 Mechanism variables

Following Coleman (15) family social capital theory, this study focused on two mediating mechanisms: the supportive pathway (perceived maternal support) and the expectation pathway (parental educational expectations).

##### 3.2.3.1 Perceived maternal support

Drawing on the stress-buffering hypothesis (26), perceived maternal support was assessed using the question: “Overall, how supportive do you think your mother is toward you?” Responses were rated on a 3-point Likert scale (1 = very supportive; 2 = somewhat supportive; 3 = not supportive). Higher scores indicate lower perceived support.

##### 3.2.3.2 Parental educational expectations

This study measured parental educational expectations as a continuous variable representing years of expected education. The original question was: “What level of education do your parents expect you to ultimately achieve?” The original ordinal responses (1 = current stage to 6 = doctoral degree) were recoded as follows: current stage = 12 years, high school/technical secondary school = 12 years, associate’s degree = 15 years, bachelor’s degree = 16 years, master’s degree = 19 years, and doctoral degree = 22 years. This transformation allows for a continuous interpretation of the effects.

#### 3.2.4 Control variables

Based on previous studies (6–8, 27), this study controlled for potential confounders, including adolescent individual characteristics (age, gender, residence, only-child status, boarding status, gifted class enrollment, and self-reported health) and family characteristics (family economic conditions and parental occupational status). Year dummies and city fixed effects were also included to account for temporal and spatial heterogeneity.

### 3.3 Data analyses & procedure

To explore the impact of maternal education on APSS, this study utilized cross-sectional data from the “Database of Youth Health” (DYH) spanning 2017 to 2020. Employing Stata 17.0 statistical software, a baseline regression model incorporating a quadratic term was constructed to examine the nonlinear relationship between the two. The robustness of the findings was further validated using U-test and instrumental variable methods.This study established the following econometric model to investigate the impact of maternal education on APSS:

(1) $$APSS_{i}=\beta_{0}+\beta_{1}Med_{i}+\beta_{2}Med_{i}^{2}+\gamma X_{i}+\mu_{c}+\lambda_{t}+\epsilon_{1_{i}}$$

where *APSS_i_* represents the level of APSS of adolescents, *Med_i_* and $Med_{\text{i}}^{2}$ represent maternal education and its square term, respectively. *X*_i_ represents the set of control variables, *μ*_c_ captures city fixed effects to account for regional characteristics that are time-invariant, *λ*_t_ represents year fixed effects, utilized to eliminate the influence of time trends, and $\epsilon_{1_{\text{i}}}$ denotes the random error term. All econometric models in this study are estimated with robust standard errors, and multicollinearity is addressed through variance inflation factor tests (VIF< 5).

## 4 Results

### 4.1 Descriptive statistics

Descriptive statistics for the main variables are provided in **Table 1**. The total scores of the SCL-90 exhibit significant individual variation (minimum = 80, maximum = 406, range = 326), indicating a marked disparity in APSS. The average years of education for mothers in the sample is 9.936 years (SD = 3.575), reflecting a diverse educational background. With reference to the SCL-90 norm for Chinese adolescents (positive criterion > 160 points), 26.15% of the sample display positive screening results on the SCL-90 (with 12.68% manifesting evident problems and 3.65% reaching a severe level). The mean SCL-90 score of the sample (131.72 ± 56.95) significantly exceeds the national norm (129.96 ± 38.76, *t* = 2.771, *p* < 0.01), indicating a critical need for targeted attention to the psychological symptom burden within this group.

**Table 1:** Descriptive statistics of variables.

Variable	Definition	Mean	Standard deviation	Minimum	Maximum	Range
Dependent variable						
APSS	Cumulative score of each item	131.7	56.95	80	406	326
Key independent variable						
Med^a^		9.936	3.575	0	19	19
Med^2^		111.5	72.56	0	361	361
Mechanism variables						
Parental educational expectations^b^	Years of expected education	15.800	2.100	12	22	10
Perceived maternal support		1.283	0.496	1	3	2
Adolescent individual characteristics						
Age		16.31	1.096	14	19	5
Gender	Male = 1; Female = 0	0.483	0.500	0	1	1
Place of residence	Urban = 1; Rural = 0	0.508	0.500	0	1	1
Gifted class^c^	Yes = 1; No = 0	0.170	0.376	0	1	1
Only-child status	Yes = 1; No = 0	0.389	0.487	0	1	1
Boarding status	Yes = 1; No = 0	0.769	0.422	0	1	1
Self-reported health status^d^		0.400	0.490	0	1	1
Family characteristics						
Family economic conditions^e^	≥3 is coded as 0	0.821	0.383	0	1	1
Father’s Employment		7.907	3.291	1	13	12
Mother’s Employment		8.570	3.209	1	13	12
Annual dummy variables		2018	1.182	2017	2020	3
City fixed effects		10.34	5.463	1	17	16

Variable processing description: Key variables were coded as follows: (1) ^a^Maternal education: Operationalized as a continuous variable based on educational level (uneducated = 0 years, primary school = 6 years, junior high school = 9 years, high school/technical secondary school = 12 years, college diploma = 15 years, bachelor’s degree = 16 years, master’s degree = 19 years); (2) ^b^Parental educational expectations: Originally measured on a 6-point ordinal scale (1 = current educational stage, 6 = doctoral degree). For the analysis, this variable was recoded into a continuous variable representing years of expected education: current stage = 12 years, high school/technical secondary school = 12 years, college diploma = 15 years, bachelor’s degree = 16 years, master’s degree = 19 years, doctoral degree = 22 years; (3) ^c^Gifted class: The original scale was 1-3 points (1 = not like this, 2 = like this), recoded into a binary variable (0 = not enrolled in a gifted class, 1 = enrolled in a gifted class); (4) ^d^Self-reported health: The original scale was 1-5 points (1 = excellent to 5 = poor), recoded into a binary variable (0 = excellent or very good, 1 = good, average, or poor); (5) ^e^Family economic status: The original scale ranged from 1 to 5 points, with higher scores indicating greater wealth, and was recoded into a binary variable [0 = medium or above (≥3 points), 1 = poor].

### 4.2 Baseline regression analysis results

This study employed a quadratic model to investigate the non-linear relationship between maternal education and APSS, with regression estimates provided in **Table 2** Model 1, which includes only the linear term, indicating a significant negative association between maternal education and SCL-90 scores (*β* = -2.246, *p* < 0.01), meaning higher maternal education is linked to lower APSS. The inclusion of the quadratic term in Model 2 yields a positive linear coefficient (*β* = 2.480, *p* < 0.05) and a significantly negative quadratic coefficient (*β* = -0.242, *p* < 0.01), providing preliminary evidence for an inverted U-shaped relationship between maternal education and APSS. This pattern persists after accounting for individual characteristics, family variables, and fixed effects in Models 3-5. The estimates from Model 5 show a linear coefficient of 3.549 (*p* < 0.01) and a quadratic coefficient of -0.275 (*p* < 0.01), with the inflection point calculated at 6.442 years (further details are provided in the “Robustness Test” section). The findings suggest that psychological symptoms peak when maternal education is 6.442 years, approximately the level of primary school graduation, thus supporting Hypothesis H1.

**Table 2:** Baseline regression analysis.

Variable	Model 1	Model 2	Model 3	Model 4	Model 5
	APSS	APSS	APSS	APSS	APSS
Med	-2.246^***^	2.480^**^	3.330^***^	3.549^***^	3.549^***^
	(0.270)	(0.966)	(0.976)	(0.955)	(0.920)
Med^2^		-0.242^***^	-0.291^***^	-0.275^***^	-0.275^***^
		(0.048)	(0.050)	(0.049)	(0.045)
Control variables	No	No	Yes	Yes	Yes
Annual dummy variables	No	No	No	Yes	Yes
City fixed effects	No	No	No	No	Yes
R^2^	0.020	0.027	0.067	0.130	0.130
R^2^_a	0.020	0.027	0.064	0.125	0.125
N	3423	3423	3423	3423	3423

The values in parentheses are robust standard errors; ***, **, and * indicate significance at the 1%, 5%, and 10% levels, respectively; same below.

### 4.3 Robustness check

#### 4.3.1 Utest test

**Figure 2:**
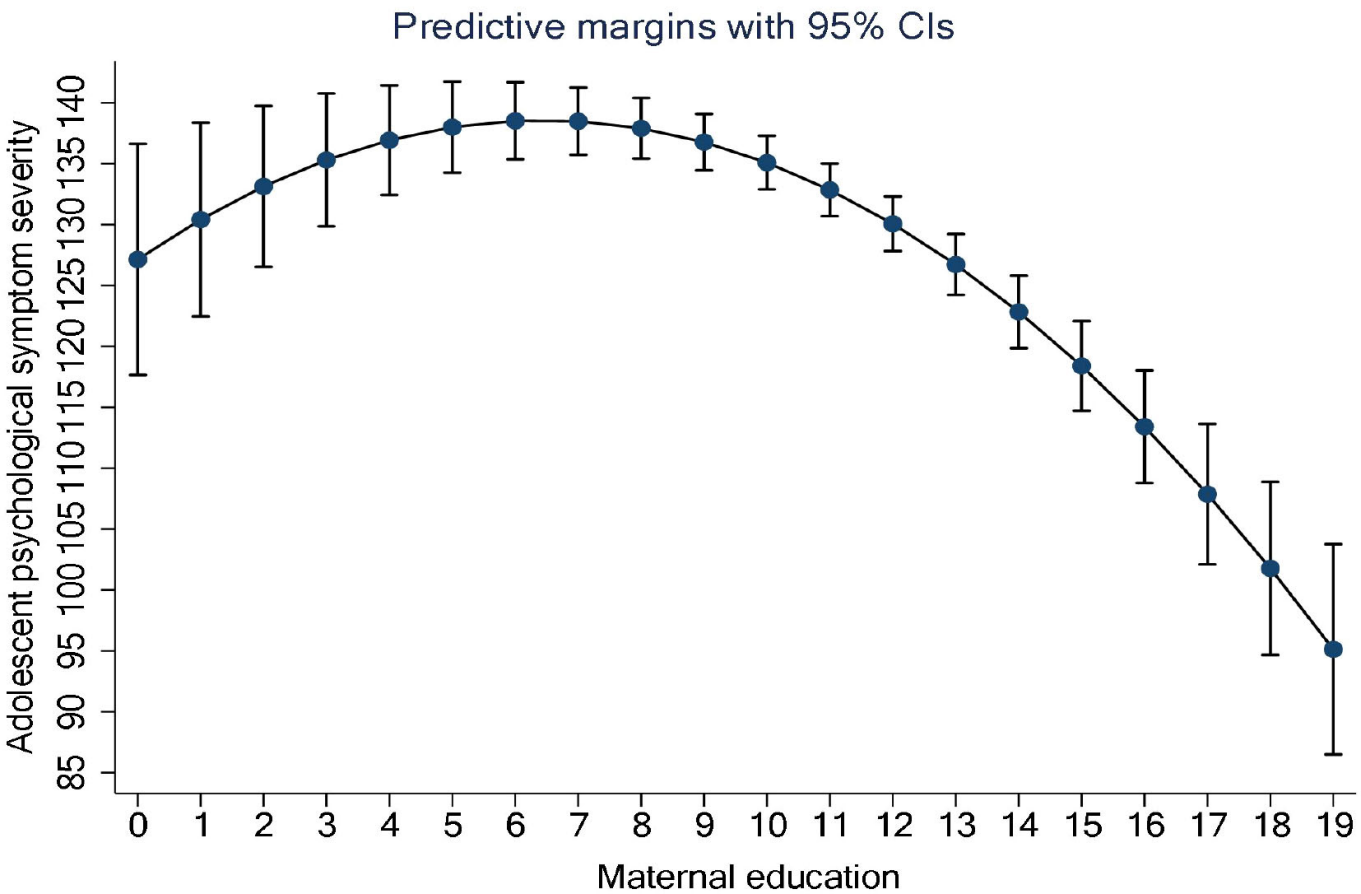
Schematic diagram of the inverted U-shaped relationship between maternal education and APSS. APSS was measured by the SCL-90; higher scores indicate greater symptom severity.

To ascertain the non-linear relationship between maternal education and APSS, this study employed the U-test method proposed by Lind and Mehlum (28) for rigorous testing. The results in **Table 3** revealed that the range of maternal education spanned 0-19 years, with a left endpoint slope of 3.549 (*p* < 0.01) and a right endpoint slope of -6.919 (*p* < 0.01). The extremum at 6.442 years was found within the data distribution (**Figure 2**). These findings not only confirmed the significance of the quadratic coefficient but also further validated the statistical significance of the inverted U-shaped relationship, thereby providing robust support for the baseline regression results.

**Table 3:** Utest results.

Variable	Lower threshold	Upper threshold
Range	0.000	19.000
Slope	3.549	-6.919
t-value	3.859	-7.687
*P*>| t |	0.000	0.000

#### 4.3.2 Instrumental variable method

Unlike randomized controlled trials (RCTs) or longitudinal designs commonly used for causal inference in psychiatry, our study employs the instrumental variable (IV) approach—a method originating in econometrics (29). The core identification strategy of the IV approach is that the instrument affects the outcome variable (e.g., APSS) only indirectly, through its effect on the endogenous variable (e.g., individual-level maternal education). Its validity hinges on the exclusion restriction assumption: after conditioning on the endogenous variable, the instrument should have no direct effect on the outcome and must not be correlated with unobserved confounders. The primary advantage of this method is its ability to mitigate omitted variable bias and reverse causality. However, its validity depends on strong assumptions, including the strength of the instrument and the plausibility of the exclusion restriction. We address these concerns below.

Our empirical context is no exception to these endogeneity concerns. APSS is a complex outcome influenced by a multitude of family and environmental factors (6), which are difficult to fully capture with observed control variables, potentially leading to omitted variable bias. Furthermore, reverse causality is a concern: adolescents’ pre-existing psychological symptoms may distort their perceptions of family interactions—for example, adolescents with more severe symptoms may perceive lower maternal support or feel greater pressure from parental expectations, thereby creating a bidirectional relationship that could confound our estimated effects.

To credibly identify the causal relationship between maternal education and APSS, we therefore adopt an IV strategy. Following Zheng (30), the “average years of education of urban mothers” (Ayeum) was selected as the IV. A first-stage F statistic of 192.24 (*p* < 0.001) (**Table 4**) confirmed its strong correlation with individual maternal education, rejecting the weak instrument hypothesis. Furthermore, to address the concern that city-level maternal education may correlate with unobserved city-level confounders, we controlled for city fixed effects in our main specification (Model 5, **Table 2**). The inverted U-shaped relationship remained robust after this adjustment (linear coefficient = 3.549, *p* < 0.05; quadratic coefficient = -0.275, *p* < 0.05), thereby supporting the plausibility of the exclusion restriction assumption.

**Table 4:** Instrument variable relevance test.

First stage regression						Shea’s partial R2	
Variable	R2	R2_a	Partial R2	Robust F statistic (2, 3401)	F test P-value	Shea’s partial R2	Shea’s adjusted partial R2
Med	0.4485	0.4451	0.1407	192.24	0.0000	0.0390	0.0325
Med2	0.4606	0.4572	0.0756	82.2829	0.0000	0.0210	0.0143

#### 4.3.3 Alternative estimation models

The robustness of the results is further confirmed using various estimation methods (**Table 5**). Using OLS regression (Model 6) as the reference, the 2SLS estimation (Model 7) reveals that the linear coefficient for maternal education rises from 3.549 to 9.500, and the quadratic coefficient shifts from -0.275 to -0.695, suggesting that the endogeneity problem results in the OLS underestimating the effect of maternal education. The estimates from LIML (Model 8) and GMM (Model 9) align closely with those from 2SLS, with no substantial increase in standard errors, indicating the robustness of the baseline regression findings.

**Table 5:** Robustness test results.

Variable	Model 6	Model 7	Model 8	Model 9
	OLS	2SLS	LIML	GMM
Med	3.549^***^	9.500^**^	9.500^**^	9.500^**^
	(0.920)	(3.959)	(3.959)	(3.959)
Med2	-0.275^***^	-0.695^**^	-0.695^**^	-0.695^**^
	(0.0449)	(0.276)	(0.276)	(0.276)
Constant	156.8^***^	160.2^***^	160.2^***^	160.2^***^
	(15.51)	(18.14)	(18.14)	(18.14)
Control variables	Yes	Yes	Yes	Yes
Annual dummy variables	Yes	Yes	Yes	Yes
City fixed effects	Yes	Yes	Yes	Yes
N	3423	3423	3423	3423
R^2^	0.130	0.105	0.105	0.105

***, **, and * indicate significance at the 1%, 5%, and 10% levels, respectively.

## 5 Further research

### 5.1 Mediation mechanism analysis

To test the hypothesized mediation mechanisms (H2a, H2b, H2c), we followed Hayes and Preacher (31) and used bootstrapping to quantify and test conditional indirect effects. Prior studies have used the same approach to test curvilinear indirect effects (e.g., 32, 33). This analytical framework is particularly suited to our research context, as it allows us to capture how the indirect effects of maternal education through perceived maternal support (M_1_) and parental educational expectations (M_2_) dynamically change across the entire continuum of maternal education—a core requirement for uncovering the trade-off mechanism proposed in H2c.

Our mediation model comprises the following system of equations. To mitigate potential multicollinearity between the linear and quadratic terms, all independent variables involved in interaction or quadratic terms were mean-centered prior to analysis (34).

(2) $$MS_{i}=\alpha_{0}+\alpha_{1}Med_{i}+\alpha_{2}Med_{i}^{2}+\gamma X_{i}+\mu_{c}+\lambda_{t}+\epsilon_{2_{i}}$$

(3) $$PE_{i}=c_{0}+c_{1}Med_{i}+\gamma X_{i}+\mu_{c}+\lambda_{t}+\epsilon_{3_{i}}$$

(4) $$APSS_{i}=b_{0}+b_{1}Med_{i}+b_{2}Med_{i}^{2}+d_{1}MS+d_{2}PE+d_{3}PE^{2}+\gamma X_{i}+u_{c}+\lambda_{t}+\epsilon_{4_{i}}$$

Based on these equations, we derived the conditional indirect effects for each mediator. Following Hayes and Preacher (31), the indirect effect in a nonlinear mediation model is not a single constant but is instead a function of the independent variable. Specifically:

For the perceived maternal support path (H2a): The conditional indirect effect is calculated as the product of the first-stage effect (the partial derivative of M_1_ with respect to X) and the second-stage effect (the coefficient of M_1_ on Y). This yields:

(5) $$\theta_{MS}=\left(\alpha_{1}+2\alpha_{2}Med_{i}\right)\times d_{1}$$

where *α_1_* and *α_2_* are the coefficients from Equation 2 and *d_1_* is the coefficient of *MS* from Equation 4. This formulation shows that θ_MS is a function of maternal education.

(6) $$\theta_{PE}=c_{1}\times \left(d_{2}+2d_{3}\times \;PE_{i}\right)$$

where *c_1_* is the coefficient from Equation 3, *d_2_* and *d_3_* are coefficients from Equation 4, *PE_i_* is the predicted value of parental expectations from Equation 3. Since *PE_i_* itself is a linear function of *Med_i_*, *θ_PE_* ultimately depends on maternal education as well. Equations 5 and 6 define these conditional indirect effects.

To test the significance of these conditional indirect effects, we employed Monte Carlo bootstrapping with 5, 000 resamples to calculate bias-corrected 95% confidence intervals (CIs) (31). We examined these indirect effects at six representative levels of maternal education: the inflection point (6.44 years), the sample mean (9.94 years), and values at one and two standard deviations both below and above the mean. This approach allowed us to comprehensively capture how the mediation mechanisms vary across the education spectrum. The results are presented in **Table 6**.

**Table 6:** Bootstrap analysis of the conditional indirect effects of maternal education via perceived maternal support and parental educational expectations.

Maternal education level (med)	Via perceived maternal support (θ_MS)	95% CI	Via parental expectations (θ_PE)	95% CI	Total ondirect effect (θ_Total)	95% CI
Low (-2 SD) ≈ 2.79 years	0.11^*^	[0.02, 0.20]	0.080^*^	[0.01, 0.16]	0.19^*^	[0.04, 0.35]
Medium-Low (-1 SD) ≈ 6.36 years	0.05^*^	[0.01, 0.10]	0.02	[-0.04, 0.08]	0.07	[-0.01, 0.15]
Inflection Point ≈ 6.44 years	0.04^*^	[0.00, 0.09]	-0.01	[-0.07, 0.05]	0.03	[-0.04, 0.10]
Medium (Mean) ≈ 9.94 years	-0.02	[-0.08, 0.04]	-0.06^*^	[-0.12, -0.01]	-0.08^*^	[-0.16, -0.01]
Medium-High (+1 SD) ≈ 13.51 years	-0.08^*^	[-0.15, -0.02]	-0.14^*^	[-0.23, -0.05]	-0.22^*^	[-0.35, -0.09]
High (+2 SD) ≈ 17.09 years	-0.15^*^	[-0.25, -0.06]	-0.20^*^	[-0.32, -0.08]	-0.35^*^	[-0.52, -0.18]

All models control for individual and family characteristics listed in **Table 1**, year dummies, and city fixed effects. Bootstrap CIs are bias-corrected and based on 5, 000 resamples. An asterisk (^*^) indicates the 95% CI does not include zero, meaning the conditional indirect effect is statistically significant at the p< 0.05 level.

#### 5.1.1 Mediation effect of perceived maternal support

The conditional indirect effect analysis (**Table 6**) demonstrates that the conditional indirect effect of maternal education on APSS via perceived maternal support (θ_MS) is not static but varies systematically across the education continuum. We find that θ_MS is positive and significant at low levels of maternal education (0.11, *p* < 0.05), becomes non-significant around the inflection point, and turns negative and significant at high levels of maternal education (-0.15, *p* < 0.05). This dynamic pattern supports hypothesis H2a.

**Figure 3:**
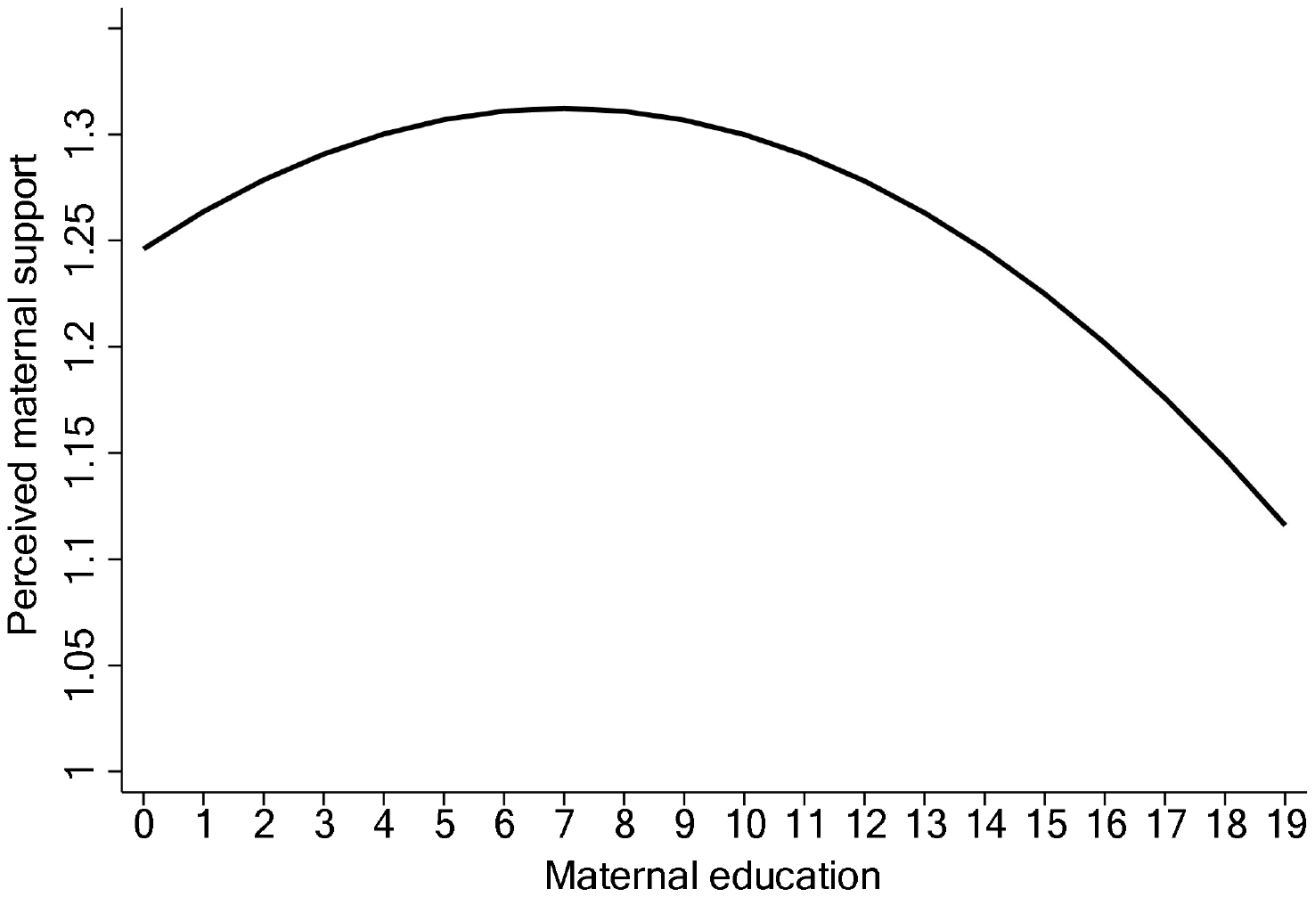
Inverted U-shaped relationship between maternal education and perceived maternal support. Perceived maternal support was measured on a 3-point scale (1 = very supportive, 2 = somewhat supportive, 3 = not supportive). Higher scores indicate poorer perceived support.

**Figure 3** elucidates the first-stage mechanism underlying this dynamic pattern. Specifically, it shows a significant inverted U-shaped relationship between maternal education and perceived maternal support. Perceived maternal support was measured on a 3-point scale (1 = very supportive, 2 = somewhat supportive, 3 = not supportive); thus, higher scores indicate lower perceived support. In **Figure 3**, the inflection point marks the highest predicted score for perceived support (i.e., the poorest perceived support). As maternal education increases beyond this point, perceived support improves (i.e., the predicted score declines).

**Figure 3** shows that perceived maternal support is poorest at intermediate levels of maternal education. This curvilinear pattern, combined with the findings that perceived support reduces APSS (9, 26, 35), explains why θ_MS shifts from a positive (risk-inducing) to a negative (protective) effect as maternal education increases.

Consequently, the mediating role of perceived maternal support transitions from a risk-inducing pathway (at low education levels, where perceived support is low) to a protective pathway (at high education levels, where perceived support is high), thereby constituting one of the two fundamental forces of the overall trade-off mechanism.

#### 5.1.2 Mediation effect of parental educational expectations

The conditional indirect effect analysis (**Table 6**) demonstrates that the conditional indirect effect of maternal education on APSS via parental educational expectations (θ_PE) also varies systematically across the education continuum. It is positive and significant at low levels of maternal education (0.08, *p* < 0.05), becomes non-significant around the inflection point, and turns negative and significant at high levels of maternal education (-0.20, *p* < 0.05). This dynamic pattern supports hypothesis H2b.

**Figure 4:**
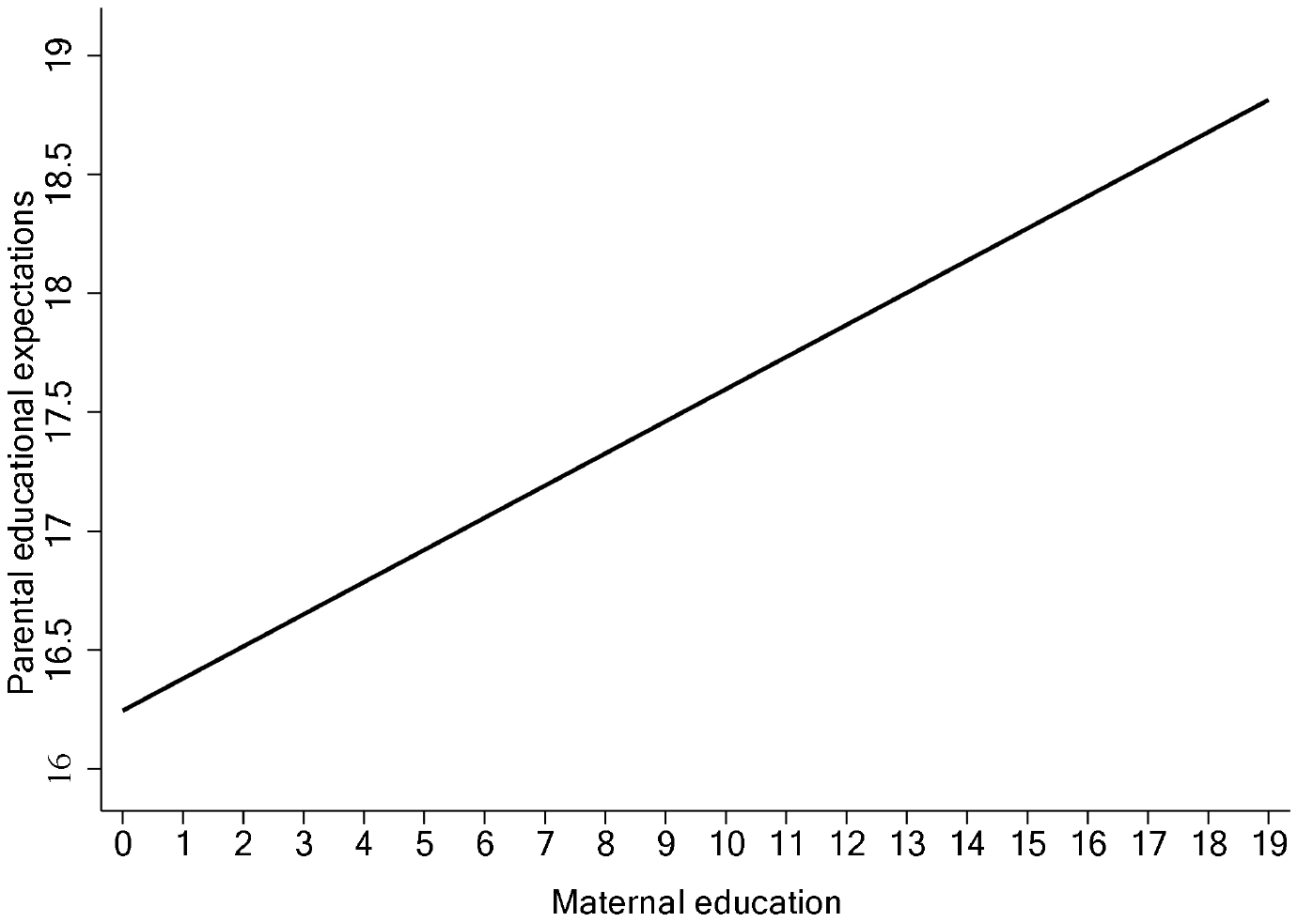
Positive linear association between maternal education and parental educational expectations. Parental educational expectations were originally measured on a 6-point ordinal scale (1 = current educational stage, 6 = doctoral degree) and recoded into years of expected education for analysis (e.g., bachelor’s degree = 16 years).

**Figure 4** elucidates the first-stage mechanism underlying this dynamic pattern. Specifically, it shows a significant positive linear relationship between maternal education and parental educational expectations. Parental expectations were measured as years of expected education; thus, higher scores indicate higher expectations. In **Figure 4**, the predicted level of expectations increases steadily with maternal education, indicating that more educated mothers hold higher expectations for their children’s educational attainment.

This positive first-stage relationship, combined with the well-documented context-dependent effect of parental educational expectations on APSS (26, 36, 37), produces the observed shift in *θ_PE_*. Specifically, at low education levels, parental educational expectations remain relatively low in absolute terms. This is often accompanied by less parent-child academic socialization communication, such as discussing the future and setting goals (17). This pattern may lead to a “parent–child consistent low expectations” developmental state, wherein adolescents lack clear goals and external norms, making them more prone to behavioral problems and low self-esteem, thereby increasing APSS (22). In contrast, at high education levels, parental expectations increase significantly. When these high expectations are effectively transmitted through academic socialization communication, they can satisfy adolescents’ basic psychological needs and enhance academic engagement, thereby directly and indirectly reducing APSS (17).

Consequently, the mediating role of parental educational expectations transitions from a risk-inducing pathway (at low education levels) to a protective pathway (at high education levels), thereby constituting the other of the two fundamental forces of the overall trade-off mechanism.

#### 5.1.3 The synergistic reversal trade-off: integrating the two pathways

The pattern of conditional indirect effects in **Table 6** provides direct quantitative evidence consistent with the hypothesized trade-off mechanism (H2c). The total indirect effect of maternal education on APSS is not constant; rather, it transitions from significantly positive to non-significant, and then to significantly negative as maternal education increases. This dynamic pattern aligns with the conceptual logic of trajectory coupling and trade-off modeling discussed in Carvalho and Ravindran (38).

Specifically, the trade-off unfolds across three distinct phases, as shown in **Table 6**.

Low Education (≤ 6.36 years, i.e., medium-low and below): Negative Trade-off. At low to medium-low levels, perceived maternal support is lower than parental educational expectations. This mismatch creates a perception of loss and loss aversion, causing both pathways to operate as risk factors. θ_MS is positive (risk-inducing), and θ_PE is also positive (risk-inducing). The total indirect effect is significantly positive (e.g., at the lowest education level-2D, θ_Total = 0.19, 95% CI [0.04, 0.35]), meaning the combined effect is risk-inducing. Neither pathway provides protection.

Near the Inflection Point (6.442 years): Balancing Point. At this point, the relative relationship between perceived maternal support and parental educational expectations approaches a dynamic equilibrium. The small positive and negative effects through each pathway effectively cancel each other out, resulting in a net indirect effect close to zero (θ_Total = 0.03, 95% CI [-0.04, 0.10]). This is the critical transition zone where the pathways begin to reverse direction.

High Education (≥ 9.94 years, i.e., at and above the mean): Protective Trade-off. As maternal education increases further, perceived maternal support now matches or exceeds parental educational expectations. This match creates a perception of gain, causing both pathways to operate as protective factors. θ_MS is negative (protective), and θ_PE is also negative (protective). The total indirect effect is significantly negative (e.g., at the mean education level, θ_Total = -0.08, 95% CI [-0.16, -0.01]), with both pathways converging to provide a strong, overall protective effect.

These findings support Hypothesis H2c. The inverted U-shaped relationship is not driven by a single static mechanism, but by the dynamic matching/mismatching between perceived maternal support and parental educational expectations, which governs the synchronous reversal of both pathways.

### 5.2 Moderating effect analysis of only-child status

To explore the heterogeneous features of the relationship between maternal education and APSS, this study introduces only-child status (Oc) as a moderator variable. Following the method of Aiken et al. (34), an expanded model incorporating interaction terms (Equation 7) is constructed to substantiate the moderating mechanism. In comparison to the baseline model (Equation 1), the expanded model systematically assesses the impact of the moderator variable on the non-linear relationship by introducing interaction terms between maternal education (linear and quadratic terms) and only-child status. To mitigate multicollinearity concerns, all higher-order terms are centered, and the specific model fitting results are presented in **Table 7**.

**Table 7:** Moderation effect regression results.

Variable	Baseline model	Model 10
	APSS	APSS
Med	3.549^**^	5.069^**^
	(0.920)	(0.913)
Med^2^	-0.275^**^	-0.310^**^
	(0.045)	(0.050)
Med×Oc		-5.079^**^
		(2.016)
Med^2^×Oc		0.162^*^
		(0.091)
Control variables	Yes	Yes
Annual dummy variables	Yes	Yes
City fixed effects	Yes	Yes
R^2^	0.130	0.134
R^2^_a	0.125	0.128
N	3423	3423

***, **, and * indicate significance at the 1%, 5%, and 10% levels, respectively.

(7) $$APSS_{i}=\delta_{0}\;+\delta_{1}Med_{i}\;+\delta_{2}Med_{i}^{2}\;+\delta_{3}Oc\;+\delta_{4}Oc\times Med_{i}+\delta_{5}Oc\times {Med}_{i}^{2}+\;\mu X_{i}+\epsilon_{i}$$

**Figure 5:**
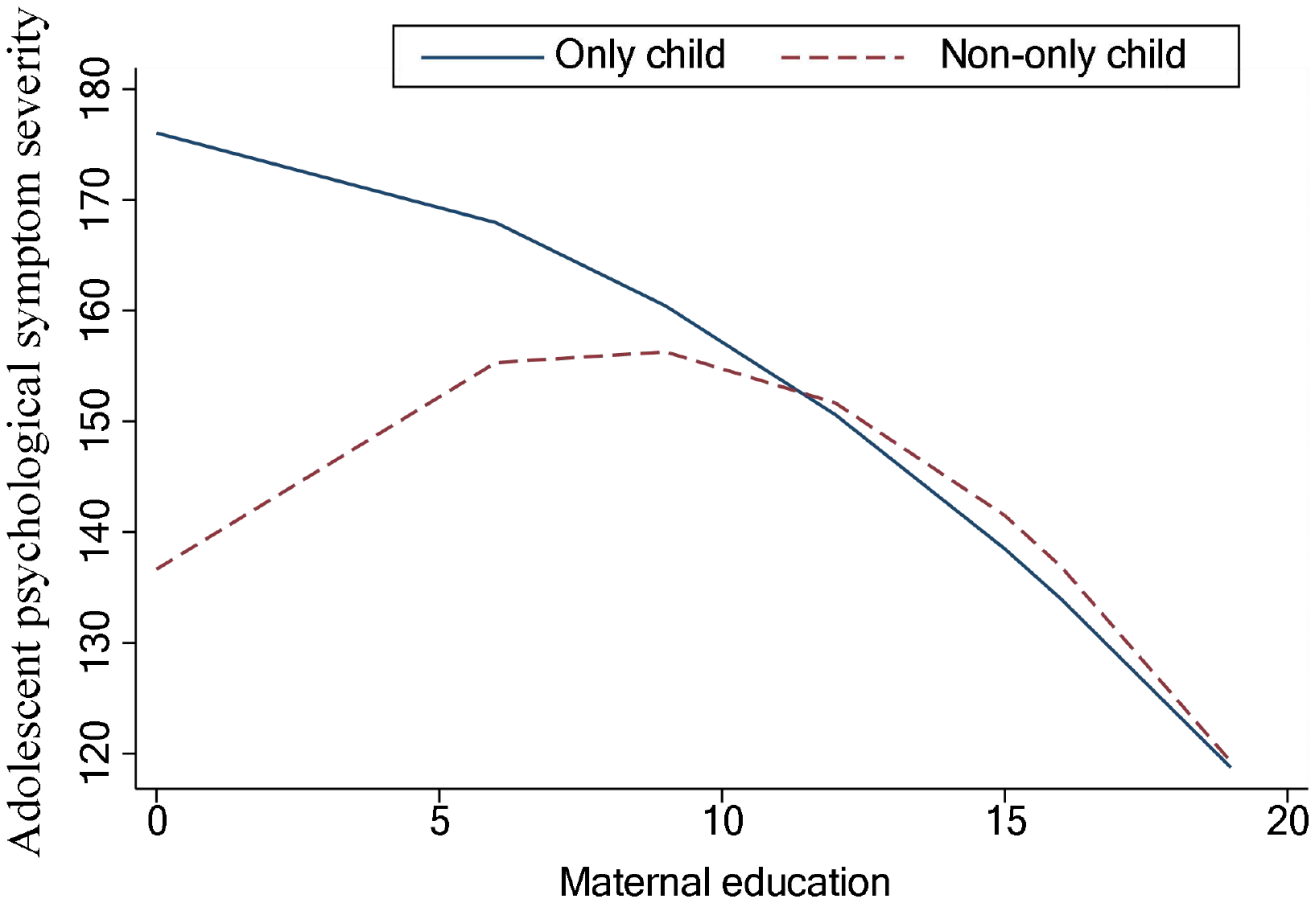
Moderating effect of only-child status.

Model 10 in **Table 7** shows that the interaction effects of only-child status on the linear (coefficient = -5.079, p < 0.05) and quadratic (coefficient = 0.162, p < 0.1) terms of maternal education are significant, suggesting only-child status moderates the inverted U-shaped relationship between maternal education and APSS. The moderation effect analysis presented in **Figure 5** reveals that: (1) with lower maternal education (< 12 years), only children have higher SCL-90 scores than non-only children, indicating that in this group, only-child status is associated with greater APSS; (2) with higher maternal education (≥ 12 years), only children’s SCL-90 scores are lower than those of non-only children, indicating that only-child status is associated with lower symptom severity under higher maternal education.

The findings support hypothesis H3, affirming that only-child status moderates the strength of the inverted U-shaped relationship between maternal education and APSS via a differentiated resource allocation mechanism.

## 6 Discussion

Research on maternal education and APSS has predominantly focused on linear effects, whereas the non-linear mechanisms underlying this relationship remain underexplored. This gap is particularly pronounced in China, where the traditional role division of “men in charge of the outside world and women in charge of the inside” and the cultural tradition of “hoping one’s child will become a dragon” shape unique family dynamics. To address this gap, this study utilizes data from the DYH (2017–2020) to test the inverted U-shaped relationship and propose a “synergistic reversal trade-off” model of its underlying micro-mechanisms.

The baseline regression results reveal an inverted U-shaped relationship between maternal education and APSS: SCL-90 scores peak at 6.442 years of maternal education and decline at both lower and higher levels. This does not mean that mothers with zero education are “better”. Rather, this inflection point at approximately 6.442 years is not where a single pathway exerts its maximum risk, but the critical equilibrium point where two proximal mechanisms—perceived maternal support and parental educational expectations—simultaneously reverse direction.

The mechanism analysis provides a micro-level explanation for this non-linear relationship. Specifically, as maternal education increases, both the supportive pathway (perceived maternal support) and the expectation pathway (parental educational expectations) simultaneously shift from risk-inducing to protective factors. This dual-pathway “synergistic reversal trade-off” is the fundamental driver of the inverted U-shaped curve. At this critical threshold (**Table 6**), the supportive pathway still presents a risk (*θ_MS_* = 0.04), while the expectation pathway has already shifted to protection (*θ_PE_* = −0.01). The near-zero total indirect effect (*θ_Total_* = 0.03) confirms that the two pathways cancel each other out at this point, marking the transition from a dual-risk to a dual-protection state. This finding offers new empirical evidence for the non-linear mechanism framework proposed by Haans et al. (39).

Our heterogeneity analysis shows that the inverted U-shaped curve is significantly steeper in only-child families and flatter in non-only-child families. This finding reflects how family resource concentration differentially amplifies the effects of the two pathways across education levels. At lower maternal education levels, only-child families lack the buffer of siblings, so the mother’s limited support and resources are concentrated entirely on one child. In this context, the pressure from low support and high expectations is highly intensified, exposing only children to greater psychological risk than non-only children. Conversely, at higher maternal education levels, the advantages of only-child families become apparent. Only children exclusively benefit from the high-quality support that educated mothers provide—such as cognitive stimulation, emotional guidance, and ample material security. In this context, high parental expectations are less likely to be perceived as pressure; instead, they are matched by available resources and a supportive environment, allowing only children to more smoothly internalize these expectations as self-motivation. Consequently, their psychological symptoms decline more rapidly at higher education levels, yielding stronger protective effects. Importantly, this inflection point is not fixed; it varies systematically with individual characteristics and environmental contexts (10, 14, 39, 40).

This study has several limitations that suggest directions for future research. First, the measurement of perceived maternal support relied on a single three-point item, which may not capture its full complexity. Future studies should employ multi-dimensional, multi-informant scales (41) to refine this pathway. Second, the cross-sectional design precludes ruling out reverse causality. While our theoretical framework posits that maternal education shapes APSS through perceived support and expectations, the opposite direction is equally plausible: adolescents’ pre-existing psychological symptoms may distort their perceptions of family interactions, thereby reducing perceived support or increasing felt expectations. Emerging evidence supports this bidirectional perspective. For instance, Bai et al. (42) documented reciprocal relations between parental autonomy support and internalizing problems among Chinese early adolescents, mediated by self-compassion. This suggests that APSS may function both as an outcome and as an antecedent of perceived parenting behaviors. Future research should adopt longitudinal cross-lagged panel designs to formally test the dynamic bidirectional relationship of the trade-off mechanism identified in this study. Third, our model assumes the two pathways operate independently, whereas their interaction could be an alternative mechanism generating the inverted U-shaped relationship. Future work could directly test this interaction using alternative identification strategies. Fourth, our instrumental variable (city-level average maternal education) may not fully satisfy the exclusion restriction. Although we include city fixed effects, time-varying city-level confounders—such as school quality and cultural norms—may still pose a threat. Thus, our IV results should be interpreted as supportive rather than definitive evidence. Future studies could employ more refined identification strategies (e.g., neighborhood/school-level instruments) to strengthen causal inference. Finally, the identified inflection point (6.442 years) is not a universal constant but a condition-dependent threshold (14). Future cross-cultural or cross-regional comparative investigations are needed to explicitly test how this inflection point shifts across different contexts (10, 39, 40).

From a global perspective, the synergistic reversal trade-off mechanism identified in this study may extend beyond the Chinese context. In societies characterized by intense educational competition and high parental expectations—such as other East Asian or Southern European countries—similar patterns of resource-expectation mismatches could emerge. Future comparative research is needed to examine how cultural and institutional contexts (e.g., social safety nets, education systems) shift the inflection point and the steepness of this inverted U-shaped relationship.

## 7 Conclusions

This study challenges the linear-benefit assumption to reveal an inverted U-shaped relationship between maternal education and APSS. The core mechanism underlying this non-linearity is a synergistic reversal trade-off between two proximal pathways: maternal support and parental expectations. This dual-pathway reversal effect is robust and amplified in only-child families.

These findings have concrete implications for policy and practice. For families with low-to-middle maternal education, interventions should focus on helping parents bridge the “resource-expectation gap” through initiatives such as parenting schools, providing them with strategies for effectively delivering support. For families with higher maternal education, policies should aim to ensure that high expectations are translated into autonomy support, thereby preventing the unintended pressure stemming from overly high expectations. Furthermore, given that the trade-off effect is significantly amplified for only children, providing targeted support for them is also crucial.

At a broader level, these findings underscore the need for family-oriented mental health policies to move beyond simply promoting maternal education. Instead, they should focus on fostering a dynamic balance between parental support and educational expectations—a principle that is likely relevant for many rapidly developing and transitional societies.

## Data Availability

Publicly available datasets were analyzed in this study. This data can be found here: https://www.ncmi.cn//phda/dataDetails.do?id=CSTR:17970.11.A0031.202107.209.V1.0.

## Footnotes

1 The adolescents in the high school stage selected for this study are in the adolescent period (12–18 years old) as defined by Erikson's eight-stage theory of psychosocial development, which is a critical period for identity exploration, academic challenges, and changing social dynamics.

2 Shandong University. Database of Youth Health. Population Health Data Archive of the National Population Health Data Center (PHDA), 2021. https://www.ncmi.cn//phda/dataDetails.do?id=CSTR:17970.11.A0031.202107.209.V1.0.
